# Algorithms for Designing Unimodular Sequences with High Doppler Tolerance for Simultaneous Fully Polarimetric Radar

**DOI:** 10.3390/s18030905

**Published:** 2018-03-18

**Authors:** Fulai Wang, Chen Pang, Yongzhen Li, Xuesong Wang

**Affiliations:** College of Electronic Science, National University of Defense Technology (NUDT), Changsha 410073, China; pangchen@nudt.edu.cn (C.P.); liyongzhen@nudt.edu.cn (Y.L.); wangxuesong@nudt.edu.cn (X.W.)

**Keywords:** radar, simultaneous polarimetric measurement, orthogonal polyphase sequences, correlation properties, Doppler tolerance

## Abstract

Simultaneous fully polarimetric radar uses orthogonal polarization channels to transmit a pair of signals, both of which must have good auto- and cross-correlation characteristics. In this paper, the design of sequences with good correlation properties and Doppler tolerance is investigated. New cyclic algorithms, namely, Cyclic Algorithm-Gradient I (CAGI) and Cyclic Algorithm-Gradient II (CAGII) are proposed to solve the optimization problem. Meanwhile, the sequences designed in this paper have ultra-low auto- and cross-correlation side-lobes in a specified lag interval. Numerical experiments are conducted to demonstrate and validate the superiority of the proposed cyclic algorithms, especially for the measurement of moving targets.

## 1. Introduction

In the past decades, polarimetric features, which can be described by a second order backscattering matrix (BSM) of the target, have been widely used in various fields, such as terrain observation, disaster surveillance and atmospheric remote sensing [1,2,3,4]. In order to accurately obtain the BSM, two fully polarimetric measurement schemes, called alternate polarimetric measurement (APM) and simultaneous polarimetric measurement (SPM) schemes have been widely investigated since the 1980s [5,6,7,8].

In alternate mode, the transmitted polarization states are switched alternately between horizontal (H) and vertical (V) polarizations while both polarizations are received simultaneously on reception. The estimations of BSM for targets with high velocity, such as satellites and spacecrafts, are inaccurate in alternate mode due to the change of their polarimetric features during the switch on transmission phase. In contrast, in simultaneous mode, the two orthogonal polarization states are transmitted and received simultaneously. Thus, the BSM of the targets can be retrieved within one pulse recurrent time (PRT). In this case, the limitation caused due to the change of the transmitted polarization states can be overcome. However, the transmitted signals must be orthogonal in order to reduce the interference caused by simultaneous transmission and reception, which is usually evaluated by the cross-correlation properties of the waveforms [9]. Numerous researchers have focused on this problem and attempted to design waveforms with good orthogonal properties. In [10], Babur et al. proposed a method for designing the quasi-orthogonal Frequency Modulated Continuous Waveforms (FMCW) and analyzed its application in multiple-input multiple-output (MIMO) radar. In addition, Liu [11] exploited the genetic algorithm to obtain the coding sequences with good correlation properties for MIMO radar applications. However, the above-mentioned algorithms have not considered the effect of the Doppler shift on the properties of the waveforms.

Designing waveforms with good autocorrelation properties is also of great importance in applications of polarimetric radar. In general, the peak of the side-lobes is related to the probability of false alarm. At the same time, if the peak of the side-lobes is high, the return of the weak targets will be masked by that of the targets with large Radar Cross Section (RCS) in nearby range cells. Therefore, the peak of the side-lobes should be as low as possible in order to improve the detection performance of weak targets. In general, there exist two major merits to measure the autocorrelation property of the waveforms that are Integrated Side-lobes Level (ISL) and Peak Side-lobes Level (PSL). For the minimization of PSL, Deng [12] designed unimodular sequences (in the rest of the article, sequences are used to denote polyphase sequences) by using the simulated annealing algorithm and analyzed their performance in applications of orthogonal netted radar. In [13], Song et al. discussed the problem of minimizing the $l_{p}$(2≤p<∞) norm on the side-lobes of autocorrelation function. They approached the minimization of PSL by increasing the value of *p*. However, their algorithm named “monotonic minimizer (MM) for $l_{p}$-metric” lacks the ability to suppress a specified part of the autocorrelation function. It should be pointed out that, in some radar applications, the maximum difference between the arrival time of the interest sequences is smaller than the duration of the emitted sequences [13,14,15,16]. Hence, only the partial side-lobes of the correlation function are needed to be low. In addition, Esmaeili-Najafabadi et al. [14] proposed a series of cyclic algorithms (CA), including PSL Minimization Quadratic Approach (PMQA), PSL minimization Algorithm where the smallest Rectangular (PMAR), PSL Optimization Cyclic Algorithm (POCA) and Randomized PSL Optimization Cyclic Algorithm (RPOCA), by using the Chebyshev distance and $l_{\infty }$ norm. They addressed the problem of optimizing the specified part of the autocorrelation function. Meanwhile, a set of waveforms with low cross-correlation side-lobes can be obtained by applying chaotic waveforms as the initial sequences in their algorithms. However, these four algorithms lack the ability to suppress the specified side-lobes for the cross-correlation function. For the minimization of ISL, P. Stoica and H. He et al. [15,16] proposed a series of cyclic algorithms for unimodular sequences, including CA-pruned (CAP), CA-new (CAN), weighted-CAN (WeCAN) and CA-direct (CAD). In addition to autocorrelation, they minimized the cross-correlation between the generated sequences and optimized the specified part of the correlation (in the rest of the article, correlation is used to denote both auto- and cross-correlation) function. However, the practical convergence rate of these algorithms becomes slow with the increase in the length of the sequences and they lack the ability to suppress all the side-lobes of the correlation function.

Furthermore, all of the above-mentioned algorithms have been proposed for the applications of static or low-velocity targets. For moving targets, Doppler loss occurs at the matched filters on reception [17]. It has been pointed out in [18] that the phase-coded sequences are quite sensitive to the Doppler shift. In other words, even if the velocity of the target is low, the output of the matched filters will still significantly deteriorate compared with the processing results of the static target’s echoes. In a recent work, an efficient gradient algorithm has been proposed to optimize the autocorrelation property of the sequences in different Doppler shift [19]. However, this algorithm cannot optimize the cross-correlation properties of the sequences, which is important for the measurement of simultaneous polarimetric radar as mentioned before. Cui et al. [20] proposed an accelerated iterative sequential optimization (AISO) algorithm that can minimize the ISL of the autocorrelation function in different Doppler shift of interest and reduce the computations compared with the algorithm in [19]. However, the AISO algorithm also does not consider the optimization of the cross-correlation properties of the sequences. In addition, Doppler tolerant complementary code sets are also developed these days because of their potential of making all the autocorrelation side-lobes sum to zero, at least in theory, but the orthogonality of the complementary sequences are not considered in most articles [21,22]. In addition, the Doppler frequency in [19,20] is considered to be a constant in one PRT, which means that the target is assumed to be in a uniform rectilinear motion state. However, the instantaneous acceleration of maneuvering targets, such as missile and spacecraft, can reach 10*g* (*g* is the gravitational acceleration). At this moment, the Doppler frequency is not a constant in one PRT, and the Doppler phase model should be modified to match the movement of the targets.

The above-mentioned three metrics are usually regarded as the measures of the sequences used in fully polarimetric radars. The low cross-correlation side-lobes improve the estimation accuracy of BSM, the low autocorrelation side-lobes increase the detection performance of weak targets and the high Doppler tolerance enhances the adaptability of the waveforms to the movement of targets. In most studies, this design problem has been usually reformulated as an optimization of a complex matrix [12,13,14,15,16,17,23]. The requirements of Doppler tolerance, autocorrelation and cross-correlation properties make the optimization problem difficult. This paper extends the approaches in [19] and proposes new cyclic algorithms for designing sequences with good correlation properties and Doppler tolerance. The rest of this paper is organized as follows: Section 2 presents the problem formulation; in Section 3, the Cyclic Algorithm-Gradient I (CAGI) and Cyclic Algorithm-Gradient II (CAGII) algorithms are introduced to design the sequences with good properties; the numerical examples are provided in Section 4 to verify the performance of the proposed algorithms, followed by the conclusions in Section 5.

Notation: Boldface uppercase and lowercase letters are used to represent matrices and vectors, respectively. See Table 1 for other notations used in this paper.

## 2. Simultaneous Polarimetric Measurement and Doppler Analysis

**Figure 1:**
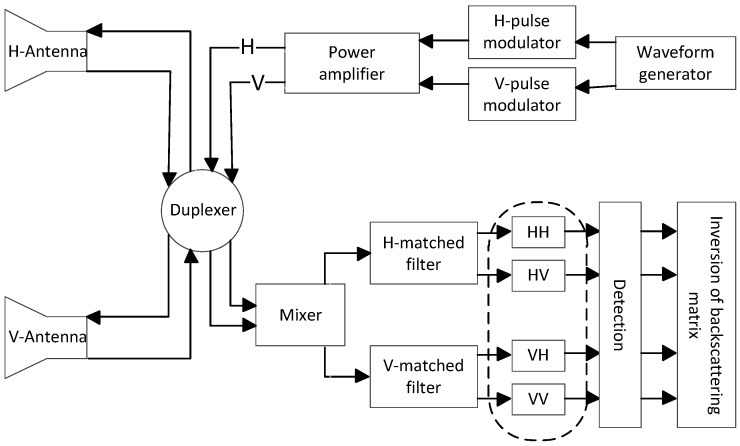
The signal processing flow chart of the simultaneous polarimetric measurement.

The simplified signal processing flow chart of the SPM is depicted in Figure 1. Let $s_{H}\left(t\right)$ and $s_{V}\left(t\right)$ represent the simultaneously transmitted signals via two orthogonal polarization channels. To facilitate the discussion, the transmitted signals can be given in vector form as:

(1) $$s\left(t\right)={\left[s_{H}\left(t\right)\;s_{V}\left(t\right)\right]}^{T}.$$

For a point target, the received signals are the Doppler-shifted and time-delayed version of the transmitted signals. Thus, they can be expressed as [24]:

(2) $$r\left(t\right)=\left[\begin{array}{c} r_{H}\left(t\right)\\ r_{V}\left(t\right) \end{array}\right]=R^{T}\cdot S\cdot T\left[\begin{array}{c} s_{H}\left(t-\tau \right)\cdot e^{j2\pi f_{d}t}\\ s_{V}\left(t-\tau \right)\cdot e^{j2\pi f_{d}t} \end{array}\right],$$

where $r_{H}\left(t\right)$ and $r_{V}\left(t\right)$ represent the received signals from the two orthogonal polarization channels, respectively, τ is the round-trip propagation delay and $f_{d}$ is the Doppler frequency determined by the target velocity and the carrier wavelength. R and T represent the effect of channel, antenna and propagation path on the BSM during the reception and transmission, respectively, and S is the BSM of the target. Processed by the matched filters shown in Figure 1, the output can be expressed as:

(3) $$\begin{array}{cc} \left[\begin{array}{c} {\hat{o}}_{\mathrm{HH}}\;\;{\hat{o}}_{\mathrm{HV}}\\ {\hat{o}}_{\mathrm{VH}}\;\;{\hat{o}}_{\mathrm{VV}} \end{array}\right] & =\left[\begin{array}{c} r_{H}\left(t\right)⊗h_{H}\left(t\right)\;\;\;r_{H}\left(t\right)⊗h_{V}\left(t\right)\\ r_{V}\left(t\right)⊗h_{H}\left(t\right)\;\;\;r_{V}\left(t\right)⊗h_{V}\left(t\right) \end{array}\right]\\ & =R^{T}\cdot S\cdot T\left[\begin{array}{c} m_{\mathrm{HH}}\;\;m_{\mathrm{HV}}\\ m_{\mathrm{VH}}\;\;m_{\mathrm{VV}} \end{array}\right], \end{array}$$

where

(4) $$h_{p}\left(t\right)=A_{p}s_{p}^{*}\left(t_{0}-t\right),$$

(5) $$m_{pq}=\left[s_{p}\left(t-\tau \right)\cdot e^{j2\pi f_{d}t}\right]⊗h_{q}\left(t\right).$$

The subscripts p,q denote the H and V polarization channels. $h_{H}\left(t\right)$ and $h_{V}\left(t\right)$ represent the matched filters of H and V polarization channels, respectively. When the transmitted signals satisfy the orthogonal condition, i.e.,

(6) $$m_{pq}=0,\;p\neq q,$$

then, if R and T are known, all the parameters of the BSM can be estimated accurately. The sequences used for simultaneous polarimetric measurement can be written as:

(7) $$\left\{\begin{array}{c} s_{H}\left(t\right)=\frac{1}{\sqrt{N\tau_{0}}}\sum_{n=1}^{N}\alpha \left[t-\left(n-1\right)\tau_{0}\right]x_{H}\left(n\right),\\ s_{V}\left(t\right)=\frac{1}{\sqrt{N\tau_{0}}}\sum_{n=1}^{N}\alpha \left[t-\left(n-1\right)\tau_{0}\right]x_{V}\left(n\right), \end{array}\right.$$

where

(8) $$x_{p}\left(n\right)=e^{j\phi_{p}\left(n\right)},\;p=H,V\;\mathrm{and}\;n=1,\cdots ,N$$

are the sequences to be designed, $\phi_{p}\left(n\right)$ can be an arbitrary value between $\left[-\pi ,\pi \right]$ (in the rest of the article, *p* and *q* are used to denote H and V), *N* is the length of the sequences, $\tau_{0}$ is the time duration of the sub-pulse and α(t) is the envelope function with unit amplitude.

The (aperiodic) cross-correlation function of ${\left\{x_{p}\left(n\right)\right\}}_{n=1}^{N}$ and ${\left\{x_{q}\left(n\right)\right\}}_{n=1}^{N}$ at lag *k* is defined as:

(9) $$\begin{array}{c} r_{pq}\left(k\right)=\sum_{n=k+1}^{N}x_{p}\left(n\right)x_{q}^{*}\left(n-k\right)=r_{qp}^{*}\left(-k\right),\\ \;k=0,\cdots ,N-1. \end{array}$$

When p=q, Equation (9) becomes the autocorrelation function of ${\left\{x_{p}\left(n\right)\right\}}_{n=1}^{N}$. It should be pointed out that, in the real radar system, each code interval has more than one point dependent on a frequency of discretization. This leads to the output of the matched filters to be different from the discrete correlation function $r_{pq}\left(k\right)$ defined in this paper. However, the authors have proved in [25] that the discrete correlation function $r_{pq}\left(k\right)$ has almost the same properties as the continuous correlation $r_{pq}\left(t\right)$, where *t* is the continuous delay. Therefore, it is reasonable to optimize the discrete correlation function instead of the continuous correlation function to obtain the sequences with good correlation properties. Moreover, the correlation functions of the sequences need to be modified because, when the target moves, the Doppler loss occurs at the matched filters on the receiving end. Here, the radial velocity and the acceleration of the target are assumed to be $v_{0}$ and *a*, respectively. Under the condition of uniformly accelerated rectilinear motion, the Doppler frequency of the received signals in different times can be written as:

(10) $$f_{d}\left(n\right)=\frac{2v(n)}{\lambda_{0}}=2\frac{v_{0}+a\frac{n-1}{N-1}N\tau_{0}}{\lambda_{0}},n=1,2,\cdots ,N,$$

where $\lambda_{0}$ is the carrier wavelength. Based on Equation (10), the Doppler phase shift of the received signals in different times can be written as:

(11) $$\phi_{d}\left(n\right)=2\pi f_{d}\left(n\right)\frac{n-1}{N-1}N\tau_{0},\;n=1,2,\cdots ,N.$$

Then, the maximum Doppler phase shift can be obtained as $\phi_{\max}=2\pi f_{d}\left(N\right)N\tau_{0}$ when *n* is equal to *N*, and the Doppler frequency is an arithmetic sequence, which means $f_{d}\left(n+1\right)-f_{d}\left(n\right)$ is a constant. Based on Equations (10) and (11), Equation (9) needs to be modified due to the existence of the Doppler phase shift as follows:

(12) $${r^{\prime }}_{pq}\left(k\right)=\left\{\begin{array}{cc} & \sum_{n=k+1}^{N}x_{p}\left(n\right){\left[x_{q}\left(n-k\right)e^{j\phi_{d}\left(n-k\right)}\right]}^{*},\;0\leq k\leq N-1,\\ & \sum_{n=1}^{N+k}x_{p}\left(n\right){\left[x_{q}\left(n-k\right)e^{j\phi_{d}\left(n-k\right)}\right]}^{*},\;-N+1\leq k<0. \end{array}\right.$$

## 3. CAGI and CAGII

### 3.1. Cyclic Algorithm for Designing Sequences under One Motion State

The sequences with good correlation properties under a certain motion state can be designed by minimizing the following criterion:

(13) $$\begin{array}{cc} \psi =\sum_{k=-N+1}^{-1} & \left({\left|{r^{\prime }}_{\mathrm{HH}}\left(k\right)\right|}^{2}+{\left|{r^{\prime }}_{\mathrm{VV}}\left(k\right)\right|}^{2}\right)+\sum_{k=1}^{N-1}\left({\left|{r^{\prime }}_{\mathrm{HH}}\left(k\right)\right|}^{2}+{\left|{r^{\prime }}_{\mathrm{VV}}\left(k\right)\right|}^{2}\right)\\ & +\sum_{k=-N+1}^{N-1}\left({\left|{r^{\prime }}_{\mathrm{HV}}\left(k\right)\right|}^{2}+{\left|{r^{\prime }}_{\mathrm{VH}}\left(k\right)\right|}^{2}\right). \end{array}$$

The first two terms of Equation (13) represent the energy of the side-lobes of the autocorrelation functions and the last term is the energy of the cross-correlation functions. Obviously, reducing the energy terms will improve the correlation properties of the sequences. According to Equation (12), another thing that should be noticed is:

(14) $${r^{\prime }}_{pp}\left(0\right)=\sum_{n=1}^{N}e^{-j\phi_{d}\left(n\right)}.$$

This means that, if the motion state of the target is given, ${r^{\prime }}_{pp}\left(0\right)$ is a constant that is not related to the designed sequences ${\left\{x_{p}\left(n\right)\right\}}_{n=1}^{N}$. Thus, the following criterion can also be optimized to design the sequences with good correlation properties:

(15) $$\psi =\sum_{k=-N+1}^{N-1}\left({\left|{r^{\prime }}_{\mathrm{HH}}\left(k\right)\right|}^{2}+{\left|{r^{\prime }}_{\mathrm{VV}}\left(k\right)\right|}^{2}+{\left|{r^{\prime }}_{\mathrm{HV}}\left(k\right)\right|}^{2}+{\left|{r^{\prime }}_{\mathrm{VH}}\left(k\right)\right|}^{2}\right).$$

Before solving the optimization problem of Equation (15), it should be emphasized that, in some radar applications, the maximum difference between the arrival times of the sequences of interest is smaller than the duration of the emitted sequences [13,14,15,16,26]. This means that only the partial side-lobes of the correlation function need to be small, instead of making all the side-lobes small. Therefore, a more proper minimization criterion than Equation (15) is given by:

(16) $$\psi =\sum_{k=-P+1}^{P-1}\left({\left|{r^{\prime }}_{\mathrm{HH}}\left(k\right)\right|}^{2}+{\left|{r^{\prime }}_{\mathrm{VV}}\left(k\right)\right|}^{2}+{\left|{r^{\prime }}_{\mathrm{HV}}\left(k\right)\right|}^{2}+{\left|{r^{\prime }}_{\mathrm{VH}}\left(k\right)\right|}^{2}\right),$$

where *P* is selected based on the prior information about the applications. In this paper, the gradient descent method is used to solve the optimization problem of Equation (16). In order to facilitate the following discussion, the transmitted sequences are denoted by:

(17) $$x_{p}={\left\{x_{p}\left(n\right)\right\}}_{n=1}^{N}={\left\{e^{j\phi_{p}\left(n\right)}\right\}}_{n=1}^{N},p=H,V.$$

Since $x_{p}$ is a complex sequence with unit envelope, the gradient can be obtained by differentiating the Equation (16) with respect to its phase. Using the chain rule of the derivative, the derivative with respect to the phase of $x_{p}$ that is written as $\frac{\partial \psi }{\partial \phi_{p}\left(n\right)}$ can be obtained by calculating the derivatives with respect to the real and the imaginary parts of $x_{p}$:

(18) ∂ψ∂ϕp(n)=∂ψ∂Rexpn·∂Rexpn∂ϕp(n)+∂ψ∂Imxpn·∂Imxpn∂ϕp(n)=−sinϕp(n)·∂ψ∂Rexpn+cosϕp(n)·∂ψ∂Imxpn=−Imxpn·∂ψ∂Rexpn+Rexpn·∂ψ∂Imxpn.

More specifically, next, $\frac{\partial \psi }{\partial \phi_{H}\left(n\right)}$ is calculated and $\frac{\partial \psi }{\partial \phi_{V}\left(n\right)}$ can be obtained by the same steps. It can be observed that $\sum_{k=-P+1}^{P+1}{\left|{r^{\prime }}_{\mathrm{VV}}\left(k\right)\right|}^{2}$ is not related to ${\left\{\phi_{H}\left(n\right)\right\}}_{n=1}^{N}$. Thus,

(19) $$\frac{\partial \psi }{\partial (\cdot )}=\sum_{k=-P+1}^{P-1}\left[\frac{\partial {\left|{r^{\prime }}_{\mathrm{HH}}\left(k\right)\right|}^{2}}{\partial (\cdot )}+\frac{\partial {\left|{r^{\prime }}_{\mathrm{HV}}\left(k\right)\right|}^{2}}{\partial (\cdot )}+\frac{\partial {\left|{r^{\prime }}_{\mathrm{VH}}\left(k\right)\right|}^{2}}{\partial (\cdot )}\right],$$

where

(20) ∂(·)=∂Re(xH(n)),∂Im(xH(n)).

As b2=Re(b)2+Im(b)2 where *b* is a complex number, it can be derived that:

(21) ∂ψ∂(·)=2∑k=−P+1P−1Re(r′HH(k))∂Re(r′HH(k))∂(·)+Im(r′HH(k))∂Im(r′HH(k))∂(·)+Re(r′HV(k))∂Re(r′HV(k))∂(·)+Im(r′HV(k))∂Im(r′HV(k))∂(·)+Re(r′VH(k))∂Re(r′VH(k))∂(·)+Im(r′VH(k))∂Im(r′VH(k))∂(·).

The details for calculating the partial derivatives with respect to Re(xH(n)) and Im(xH(n)) are provided in Appendix A. Here, the results are given by Equations (22) and (23):

(22) ∂ψ∂Re(xH(n))=2∑k=−P+1P−1Rer′HH(k)xH(n−k)ejϕd(n−k)+Rer′HH(k)xH(n+k)e−jϕd(n)∗+Rer′HV(k)xV(n−k)ejϕd(n−k)+Rer′VH(k)xV(n+k)e−jϕd(n)∗,

(23) ∂ψ∂Im(xH(n))=2∑k=−P+1P−1Imr′HH(k)xH(n−k)ejϕd(n−k)−Imr′HH(k)xH(n+k)e−jϕd(n)∗+Imr′HV(k)xV(n−k)ejϕd(n−k)−Imr′VH(k)xV(n+k)e−jϕd(n)∗.

By substituting Equations (22) and (23) into the chain rule Equation (18), it can be derived that:

(24) ∂ψ∂ϕH(n)=−2ImxH(n)∑k=−P+1P−1r′HH(k)xH(n−k)ejϕd(n−k)∗−∑k=−P+1P−1r′HH(k)xH(n+k)e−jϕd(n)∗+∑k=−P+1P−1r′HV(k)xV(n−k)ejϕd(n−k)∗−∑k=−P+1P−1r′VH(k)xV(n+k)e−jϕd(n)∗.

The $\frac{\partial \psi }{\partial \phi_{V}\left(n\right)}$ can be obtained following the same procedures from Equations (19)–(24). Thus, generally, the gradient of ψ with respect to $\phi_{p}\left(n\right)$, which is the phase of the sequence, can be written as:

(25) ∂ψ∂ϕp(n)=−2Imxp(n)∑k=−P+1P−1r′pp(k)xp(n−k)ejϕd(n−k)∗−∑k=−P+1P−1r′pp(k)xp(n+k)e−jϕd(n)∗+∑k=−P+1P−1r′pq(k)xq(n−k)ejϕd(n−k)∗−∑k=−P+1P−1r′qp(k)xq(n+k)e−jϕd(n)∗,p≠q.

In order to facilitate the following discussion, ${r^{\prime }}_{pq}={\left\{{r^{\prime }}_{pq}\left(n\right)\right\}}_{n=-P+1}^{P-1}$ and $D={\left\{\phi_{d}\left(n\right)\right\}}_{n=1}^{N}$ are defined herein. Then, Equation (25) can be rewritten as:

(26) ∂ψ∂ϕp(n)=−2Imxp(n)r′pp∗⊗xp⊙Dn−ejϕd(n)r′ppr⊗xpn+r′pq∗⊗xq⊙Dn−ejϕd(n)r′qpr⊗xqn,

by defining:

(27) $$\chi ={\left\{{\left({r^{\prime }}_{pp}^{*}⊗\left(x_{p}⊙D\right)+{r^{\prime }}_{pq}^{*}⊗\left(x_{q}⊙D\right)\right)}_{n}-e^{j\phi_{d}\left(n\right)}{\left({r^{\prime }}_{pp}^{r}⊗x_{p}+{r^{\prime }}_{qp}^{r}⊗x_{q}\right)}_{n}\right\}}_{n=1}^{N}$$

Equation (26) can be expressed in a vector form:

(28) ∇p=∂ψ∂ϕp(n)n=1N=−2Imxp⊙χ.

It is well known that the convolution operation in the time domain corresponds to the product operation in the frequency domain. If $F\left(\cdot \right)$ denotes the Fast Fourier Transforms (FFT) operation, it can be derived that the convolution of a *M*-dimensional sequence b and a *N*-dimensional sequence d is equivalent to:

(29) $$b⊗d=F^{-1}\left(F\left(b^{\prime }\right)F\left(d^{\prime }\right)\right),$$

where

(30) $$b^{\prime }=\left[b\;\underset{N-1}{\underset{︸}{0\cdots 0}}\right]$$

and

(31) $$d^{\prime }=\left[d\;\underset{M-1}{\underset{︸}{0\cdots 0}}\right].$$

Therefore, Equation (29) provides an efficient computation of the gradient Equation (28), which is expressed as a sum of convolution operations. Then, by using the gradient $\nabla_{p}$, the following algorithm shown in Table 2, named the Cyclic Algorithm-Gradient I (CAGI), can be performed to obtain the sequences with good correlation properties under one certain motion state.

### 3.2. Cyclic Algorithm for Designing Sequences under a Set of Motion States

In this subsection, a new cyclic algorithm is developed to design sequences with good correlation properties for a set of motion states. Here, matrix L is used to describe the set of motion states and is defined as:

(32) $$L={\left[\begin{array}{cccc} v_{0,1} & v_{0,2} & \cdots & v_{0,L}\\ a_{1} & a_{2} & \cdots & a_{L} \end{array}\right]}_{L\times 2}^{T}$$

Obviously, each row of the matrix L represents one motion state with a velocity and acceleration of $v_{0,l}$ and $a_{l}$, respectively, where 1≤l≤L. Then, the criterion for designing sequences with good correlation properties for a set of motion states can be written as:

(33) $$\bar{\psi }=\sum_{l=1}^{L}\psi_{l}=\sum_{l=1}^{L}\sum_{k=-P+1}^{P-1}\left({\left|{r^{\prime }}_{\mathrm{HH},l}\left(k\right)\right|}^{2}+{\left|{r^{\prime }}_{\mathrm{VV},l}\left(k\right)\right|}^{2}+{\left|{r^{\prime }}_{\mathrm{HV},l}\left(k\right)\right|}^{2}+{\left|{r^{\prime }}_{\mathrm{VH},l}\left(k\right)\right|}^{2}\right)$$

where

(34) $${r^{\prime }}_{pq,l}\left(k\right)=\left\{\begin{array}{cc} & \sum_{n=k+1}^{N}x_{p}\left(n\right){\left[x_{q}\left(n-k\right)e^{j\phi_{d,l}\left(n-k\right)}\right]}^{*},\;0\leq k\leq N-1\\ & \sum_{n=1}^{N+k}x_{p}\left(n\right){\left[x_{q}\left(n-k\right)e^{j\phi_{d,l}\left(n-k\right)}\right]}^{*},\;-N+1\leq k<0 \end{array}\right.$$

and

(35) $$\phi_{d,l}\left(n\right)=2\pi f_{d,l}\left(n\right)\frac{n-1}{N-1}N\tau_{0}=4\pi N\tau_{0}\frac{\left(n-1\right)\left(N-1\right)v_{0,l}+a_{l}{\left(n-1\right)}^{2}N\tau_{0}}{{\left(N-1\right)}^{2}\lambda_{0}},n=1,\cdots N.$$

Equation (33) represents the energy of the side-lobes under different motion states of interest. Similarly, the sequences with good correlation properties under the motion states of interest can be obtained by reducing the energy of the side-lobes. According to the above discussion, it is obvious that the gradient of $\bar{\psi }$ with respect to the phase of the sequences is:

(36) $$\frac{\partial \bar{\psi }}{\partial \phi_{p}\left(n\right)}=\sum_{l=1}^{L}\frac{\partial \psi_{l}}{\partial \phi_{p}\left(n\right)}$$

where $\frac{\partial \psi_{l}}{\partial \phi_{p}\left(n\right)}$ can be calculated by Equations (18)–(28). Apparently, the gradient of $\bar{\psi }$ consists on a sum of the gradient of ψ under different motion states. Therefore, Equation (36) can also be efficiently calculated by using the FFT. By defining:

(37) $${\bar{\nabla }}_{p}=\sum_{l=1}^{L}\nabla_{p,l}=\sum_{l=1}^{L}{\left\{\frac{\partial \psi_{l}}{\partial \phi_{p}\left(n\right)}\right\}}_{n=1}^{N}$$

the following algorithm shown in Table 3, named the Cyclic Algorithm-Gradient II (CAGII), can be performed to obtain the sequences with good correlation properties under a set of motion states of interest.

## 4. Simulation Results and Discussion

In this section, the proposed algorithms are evaluated using simulations. Subsequently, some metrics are defined herein in order to compare the correlation properties. The normalized correlation is defined according to

(38) $$\begin{array}{cc} Normalized\;Correlation & =20\log10\frac{\left|{r^{\prime }}_{pq}\left(k\right)\right|}{\left|{r^{\prime }}_{pp}\left(0\right)\right|},\\ k & =0,\cdots ,N-1. \end{array}$$

When p=q, Equation (38) is the normalized autocorrelation, and, when p≠q,, it becomes the normalized cross-correlation. Afterwards, the Peak Correlation Level (PCL) and the Isolation (*I*) are:

(39) $$PCL=20\log10\frac{\max\left[\left|{r^{\prime }}_{pp}\left(i\right)\right|\right]}{\left|{r^{\prime }}_{pp}\left(0\right)\right|},i\in \left[-P+1,\cdots ,-1,1,\cdots ,P-1\right],$$

(40) $$\begin{array}{c} I=20\log10\frac{\max\left[\left|{r^{\prime }}_{pq}\left(m\right)\right|\right]}{\left|{r^{\prime }}_{pp}\left(0\right)\right|},p\neq q\;\mathrm{and}\;m\in \left[-P+1,\cdots ,-1,0,1,\cdots ,P-1\right]. \end{array}$$

### 4.1. Suppressing Specified Parts of the Correlation Functions without Doppler Shift

The performance of the proposed CAGI algorithm and the cyclic algorithm WeCAN in [16] are assessed under a scenario where the target of interest is stationary, which means $v_{0}$ and *a* in Equation (10) are zero. In particular, consider the example given as follows:

(41) $$\left\{\begin{array}{c} N=100,\\ P=20. \end{array}\right.$$

**Figure 2:**
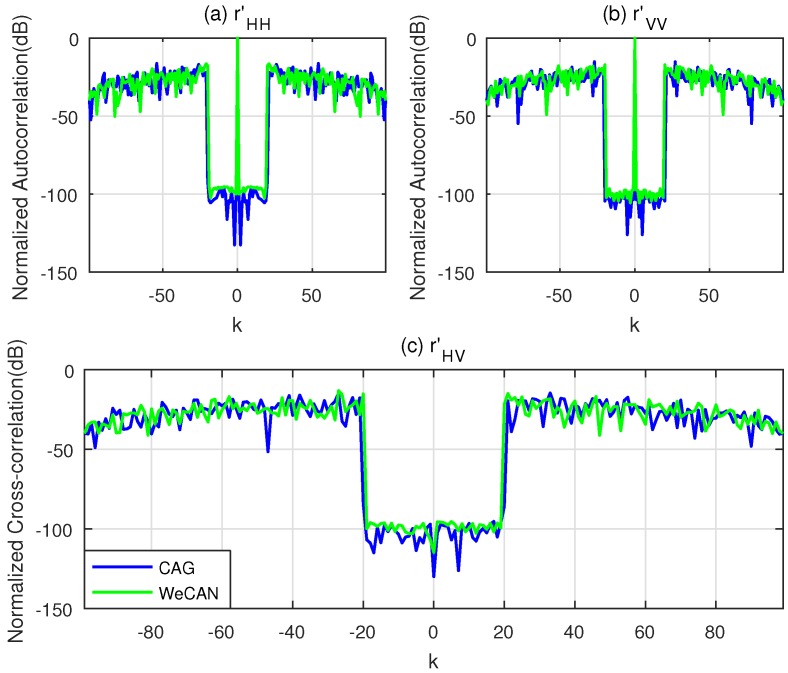
Normalized correlation of CAGI vs. WeCAN under the condition of Equation (41). (**a**) The Normalized Autocorrelation of **x**_H_; (**b**) The Normalized Autocorrelation of **x**_V_; (**c**) The Normalized Cross-correlation of **x**_H_ and **x**_V_.

All of the following simulations are implemented through Matlab 2016b (MathWorks, Natick, State of Massachusetts, the United States) on an i5 3.2 GHz machine (Intel Corporation, Santa Clara, State of California, the United States) with a 4 GB RAM. The comparison between the normalized correlation of CAGI and WeCAN is presented in Figure 2 for parameter ε = 10^−6^. It can be seen from the figure that both algorithms can suppress the side-lobes to almost zero in a specified lag interval. The PCL and *I* of the sequences designed by the two algorithms are about −100 dB, which is low enough for the applications of simultaneous polarimetric radar [28]. The main difference between the two algorithms is the consumed time. The WeCAN algorithm consumed over 1 h while the proposed CAGI algorithm only consumed 78.12 s to generate this figure. The reason is the efficient computation of the gradient by using the FFT for CAGI algorithm.

### 4.2. Suppressing Specified Parts of the Correlation Functions for One Motion State

**Figure 3:**
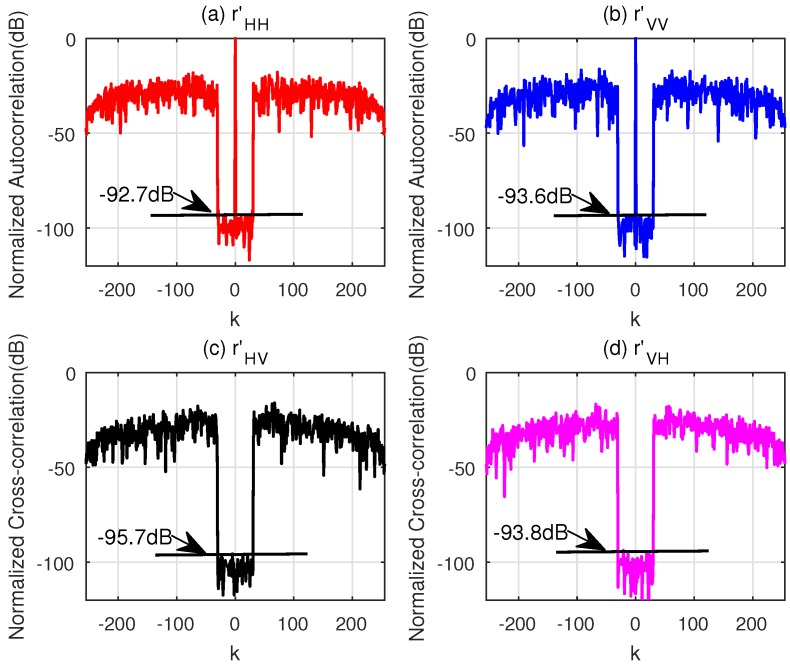
Normalized correlation of CAGI for $v_{0}$ = 3 km/s and a = 100 m/s^2^. (**a**) The Normalized Autocorrelation of **x**_H_; (**b**) The Normalized Autocorrelation of **x**_V_; (**c**) The Normalized Cross-correlation of **x**_H_ and **x**_V_; (**d**) The Normalized Cross-correlation of **x**_V_ and **x**_H_.

**Figure 4:**
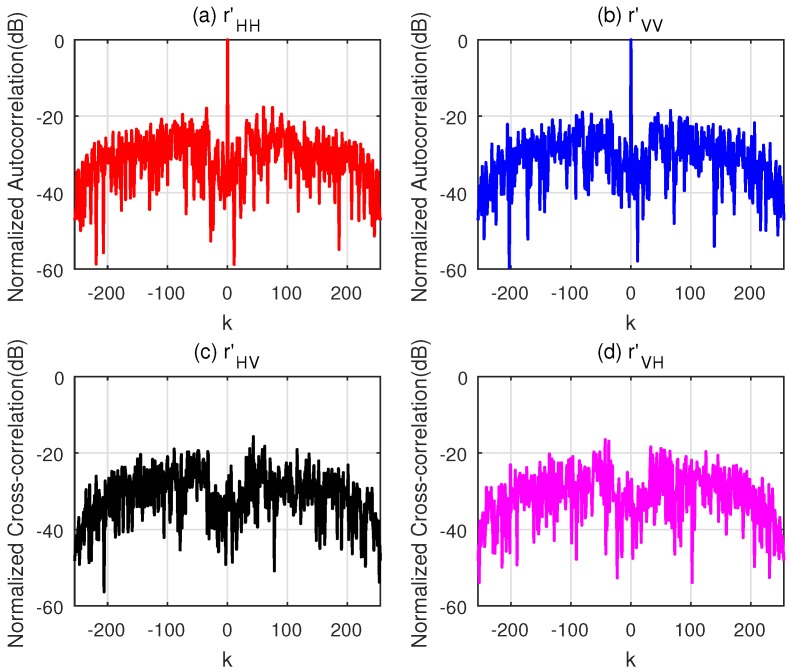
Normalized correlation of WeCAN for $v_{0}$ = 3 km/s and a = 100 m/s^2^. (**a**) The Normalized Autocorrelation of **x**_H_; (**b**) The Normalized Autocorrelation of **x**_V_; (**c**) The Normalized Cross-correlation of **x**_H_ and **x**_V_; (**d**) The Normalized Cross-correlation of **x**_V_ and **x**_H_.

The performance of the CAGI and the WeCAN algorithms are again assessed under the second scenario, where suppressing a specified part of the correlation functions under a certain motion state is considered. Here, the highly maneuvering targets, such as satellites and spacecrafts, are of concern and the simulation parameters are set in Table 4:

The normalized correlation functions of the sequences designed by the two algorithms for parameter ε = 10^−6^ are illustrated in Figure 3 and Figure 4. Obviously, the CAGI sequences provide much lower PCL and *I* compared with the sequences designed by the WeCAN algorithm. A fundamental reason is that the velocity and the acceleration of the target are considered in the objective function of the CAGI. In addition, the execution time, the iteration number, PCL and *I* (here, PCL and *I* are the average values of the sequences $x_{H}$ and $x_{V}$) are shown in Table 5. It can be easily observed from the table that the execution time per iteration of CAGI algorithm is shorter than that of the WeCAN algorithm. The reason is that the use of the FFT improves the computation efficiency of the gradient. At the same time, the CAGI algorithm takes much less iterations than that of WeCAN algorithm. Thus, the total execution time is about five percent in comparison with that of the WeCAN algorithm.

**Figure 5:**
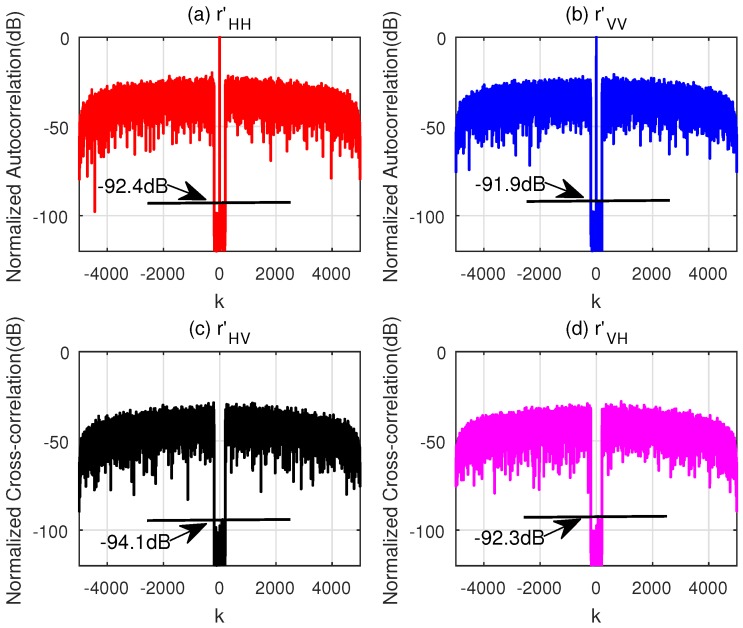
Normalized correlation of CAGI for $v_{0}$ = 1 km/s and a = 100 m/s^2^. (**a**) The Normalized Autocorrelation of **x**_H_; (**b**) The Normalized Autocorrelation of **x**_V_; (**c**) The Normalized Cross-correlation of **x**_H_ and **x**_V_; (**d**) The Normalized Cross-correlation of **x**_V_ and **x**_H_.

Another drawback of the WeCAN is that it cannot easily cope with large length of sequences (i.e., the lengths more than N=1000). In these situations, the CAGI algorithm can be applied. Table 6 provides the simulation parameters for the CAGI to optimize the sequences with length N=5000. The normalized correlation for the sequences with parameter ε = 10^−6^ are shown in Figure 5. The PCL and *I* of the sequences are about −93 dB, which are almost equal to that of the sequences with short length shown in Figure 3.

### 4.3. Suppressing Specified Parts of the Correlation Functions for a Set of Motion States

In this subsection, another situation is considered, where suppressing a specified part of the correlation functions under a set of motion states is of concern. Similarly, highly maneuvering targets, such as satellites and spacecrafts, are considered. Assume that the velocity and the acceleration of the target are within $v\in \left(1000,2000\right)$ m/s and a∈(75,150) m/s^2^, respectively. In order to facilitate the calculation, the velocity and the acceleration intervals are uniformly discretized with the grid size Δv = 10 m/s and Δa = 5 m/s^2^, respectively. Then, the matrix L that describes the motion states of interest can be written as:

(42) $$L={\left[\begin{array}{ccccccccc} 1000 & 1010 & \cdots & 2000 & \cdots \cdots & 1000 & 1010 & \cdots & 2000,\\ 75 & 75 & \cdots & 75 & \cdots \cdots & 150 & 150 & \cdots & 150, \end{array}\right]}^{T}$$

**Figure 6:**
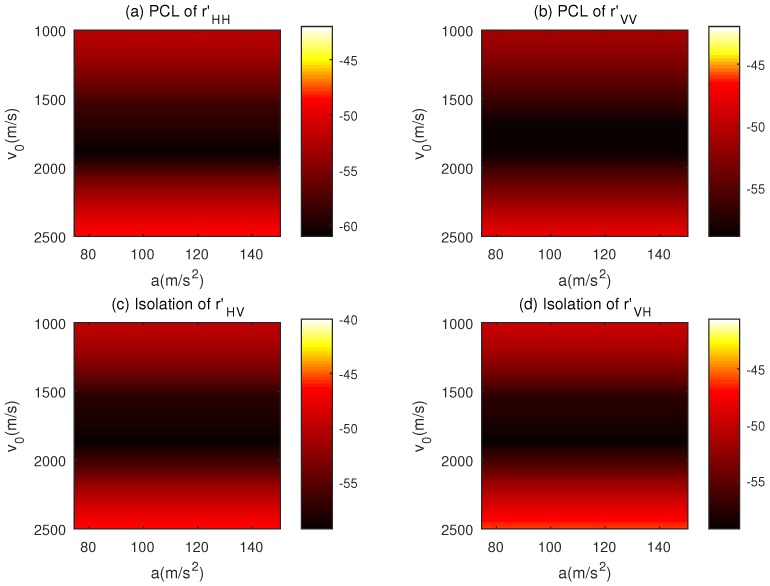
Correlation properties of the sequences designed by CAGII under the condition of Equation (42). (**a**) The PCL of the Normalized Autocorrelation $r_{\mathrm{HH}}^{\prime }$; (**b**) The PCL of the Normalized Autocorrelation $r_{\mathrm{VV}}^{\prime }$; (**c**) The Isolationof the Normalized Cross-correlation $r_{\mathrm{HV}}^{\prime }$; (**d**) The Isolation of the Normalized Cross-correlation $r_{\mathrm{VH}}^{\prime }$.

Moreover, $\lambda_{0}$ = 0.03 m, $\tau_{0}$ = 5 × 10^−9^ s, N=256 and P=30 are set for the simulations. Utilizing the proposed CAGII algorithm, the sequences with good correlation properties under a set of motion states can be obtained. Figure 6a–d illustrate the correlation properties of the sequences designed by the CAGII algorithm with parameter ε = 10^−6^. It can be seen clearly that within the given velocity and acceleration interval, the PCL and *I* of the sequences are under −50 dB, and the fluctuation of the correlation properties is within 5 dB. The reason is that the object function Equation (33) of the CAGII is the weighted sum of correlation functions with different motion states, and the weighting coefficients are all equal to 1. It can also be observed that the correlation properties of the sequences change slightly in this situation with the increasing of the acceleration. This behavior is attributed to the fact that the time length of the sequence, which is equal to the product of *N* and $\tau_{0}$, is short. Thus, the change of the velocity is unapparent in the duration of the sequences. However, when the sequence length is increased or the bandwidth of the sequence is reduced, meaning the duration of the sequence becomes longer, the change of the velocity will be obvious within one PRT of the transmitted sequences, and, accordingly, the Doppler frequency between the sub-pulse of the sequences will be different significantly.

**Figure 7:**
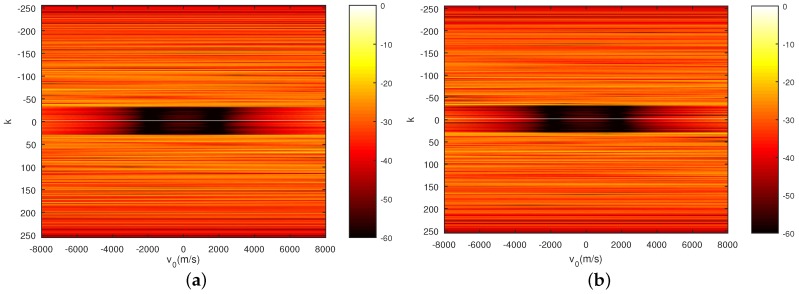
Auto-ambiguity function of the sequences with a = 100 m/s^2^. (**a**) AF of $x_{H}$ with a = 100 m/s^2^; (**b**) AF of $x_{V}$ with a = 100 m/s^2^.

**Figure 8:**
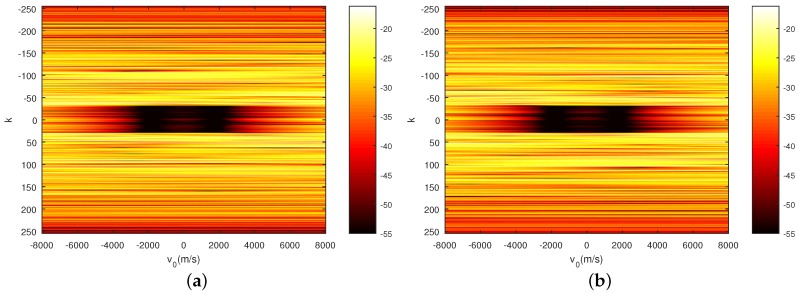
Cross-ambiguity function of the sequences with a= = 100 m/s^2^. (**a**) CF of $x_{H}$ and $x_{V}$ with a = 100 m/s^2^; (**b**) CF of $x_{V}$ and $x_{H}$ with a = 100 m/s^2^.

**Figure 9:**
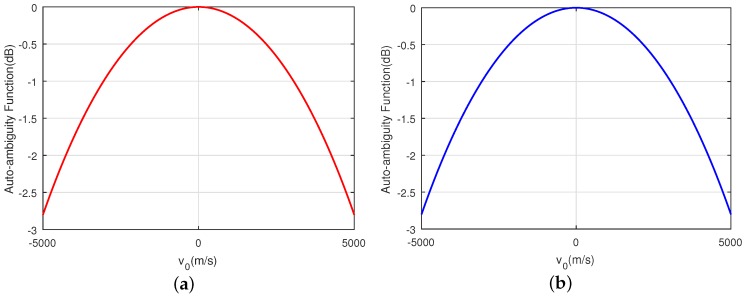
The zero-distance cut of the AF for $x_{H}$ and $x_{V}$. (**a**) AF of $x_{H}$ for zero-distance cut; (**b**) AF of $x_{V}$ for zero-distance cut.

Furthermore, in order to verify the performance of the sequences shown in Figure 6, the auto-ambiguity functions (AF) and the cross-ambiguity functions (CF) for the sequences $x_{H}$ and $x_{V}$ with acceleration a = 100 m/s^2^ are shown in Figure 7, Figure 8 and Figure 9, respectively. It can be seen that the PCL and *I* in the area of interest that refers to the area with $k\in \left[-29,29\right]$ and $v_{0}\in \left(1000,2000\right)$ m/s of the ambiguity function, is lower than −50 dB. In addition, it should be noticed from Figure 9 that the fluctuation of the peak of the normalized autocorrelation function caused by the mismatch of the Doppler loss is within 3 dB when the velocity of the target changes from v = 0 m/s to v = 5000 m/s. This phenomenon further confirms that the sequences designed by the CAGII algorithm have high Doppler tolerance under the given motion states.

## 5. Conclusions

In this paper, two new cyclic algorithms, called Cyclic Algorithm-Gradient I (CAGI) and Cyclic Algorithm-Gradient II (CAGII), are proposed for designing a pair of unimodular sequences for simultaneous polarimetric radar. The CAGI approach can design sequences with both good auto- and cross-correlation properties under one motion state. The CAGII is an extension of the CAGI and can solve the optimization problem under a set of motion states. The application of the FFT in the CAGI and the CAGII algorithms enable both approaches to obtain quite long sequences with good correlation properties. Several numerical simulations and comparative analysis are conducted to demonstrate and validate the superior performance of the sequences designed using the proposed methods compared with those obtained using other algorithms in the literature. Furthermore, the authors plan to conduct research on the extension of the proposed algorithms to the finite alphabet phase case and improve the Doppler tolerance and orthogonality of the complementary coded sequences in future.

## Figures and Tables

**Table 1:** Notations.

$a^{*}$:	the complex conjugate of a scalar *a*
Re(a):	the real part of a scalar *a*
Im(a):	the imaginary part of a scalar *a*
$a^{r}$:	the reverse of a vector a
$A^{*}$:	the complex conjugate of a matrix A
$A^{T}$:	the transpose of a matrix A
$A^{H}$:	the conjugate transpose of a matrix A
A⊙B:	the Hadamard product of two matrices A and B with the same dimension
$\left|a\right|$:	the modulus of a complex number *a*
⊗:	the convolution operation between two signals

**Table 2:** Steps for the CAGI Algorithm.

**Step 0**: Set ${\left\{x_{H}\left(n\right)\right\}}_{n=1}^{N}$ and ${\left\{x_{V}\left(n\right)\right\}}_{n=1}^{N}$ to initial sequences (e.g., ${\left\{x_{p}\left(n\right)\right\}}_{n=1}^{N}$ can be set to ${\left\{e^{j\phi_{p}\left(n\right)}\right\}}_{n=1}^{N}$, where ${\left\{\phi_{p}\left(n\right)\right\}}_{n=1}^{N}$ are independent random variables distributed in $\left[0,2\pi \right])$. Fix the motion state, which means $v_{0}$ and *a* should be given, and compute the vector ***D*** by Equation (11). **Step 1**: Calculate the gradient $\nabla_{p}$ according to Equation (28) **Step 2**: Renew the phases of the sequences using $x_{p}^{i+1}=x_{p}^{i}\cdot e^{-j\beta^{i}\nabla_{p}}$, where the step length $\beta^{i}$ is computed according to the line search algorithm [27]. **Step 3**: Repeat Steps1 and 2 until a stop criterion is satisfied, e.g., $\left|\psi^{i+1}-\psi^{i}\right|\leq \varepsilon$, where $\psi^{i}$ is the objective function at the *i*th iteration and ε is a predefined threshold.

**Table 3:** Steps for the CAGII Algorithm.

**Step 0**: Set ${\left\{x_{H}\left(n\right)\right\}}_{n=1}^{N}$ and ${\left\{x_{V}\left(n\right)\right\}}_{n=1}^{N}$ to initial sequences (e.g., ${\left\{x_{p}\left(n\right)\right\}}_{n=1}^{N}$ can be set to ${\left\{e^{j\phi_{p}\left(n\right)}\right\}}_{n=1}^{N}$, where ${\left\{\phi_{p}\left(n\right)\right\}}_{n=1}^{N}$ are independent random variables distributed in $\left[0,2\pi \right])$. Fix the set of motion states, which means the matrix ***L*** should be given. **Step 1**: Calculate the gradient ${\bar{\nabla }}_{p}$ according to Equations (28) and (37). **Step 2**: Renew the phases of the sequences using $x_{p}^{i+1}=x_{p}^{i}\cdot e^{-j\beta^{i}{\bar{\nabla }}_{p}}$, where the step length $\beta^{i}$ is computed according to the line search algorithm [27]. **Step 3**: Repeat Steps1 and 2 until a stop criterion is satisfied, e.g., $\left|{\bar{\psi }}^{i+1}-{\bar{\psi }}^{i}\right|\leq \varepsilon$, where ${\bar{\psi }}^{i}$ is the objective function at the *i*th iteration and ε is a predefined threshold.

**Table 4:** Simulation parameters.

*N*	*P*	$\lambda_{0}$	$\tau_{0}$	$v_{0}$	*a*
256	30	0.03 m	5 × 10^−9^ s	3 km/s	100 m/s^2^

**Table 5:** Comparison between CAGI and WeCAN.

	PCL	*I*	Iteration Number	Total Execution Time	Execution Time per Iteration
CAGI	−93.15 dB	−94.75 dB	6546	1093.3 s	0.168 s
WeCAN	−25.73 dB	−24.15 dB	123,521	23,047.5 s	0.187 s

**Table 6:** Simulation parameters.

*N*	*P*	$\lambda_{0}$	$\tau_{0}$	$v_{0}$	*a*
5000	200	0.03 m	5 × 10^−9^ s	1 km/s	100 m/s^2^

## Appendix A. Details of Gradient Calculation

As it has been described in Section 3, the gradient calculation is related to the calculations of the derivative with respect to the real and the imaginary parts of $x_{p}$. Here, we continue the derivation after Equation (21). Furthermore, when k≥0 (the procedures of k<0 are the same as that of k≥0), the real and the imaginary parts of ${r^{\prime }}_{\mathrm{HH}}\left(k\right)$ can be developed as follows:

(A1) Re(r′HH(k))=Re∑m=k+1NxH(m)xH(m−k)ejϕd(m−k)∗=∑m=k+1m≠n;m≠n+kNRexH(m)xH(m−k)ejϕd(m−k)∗+RexH(n)xH(n−k)ejϕd(n−k)∗+RexH(n+k)e−jϕd(n)xH∗(n)

and

(A2) Im(r′HH(k))=Im∑m=k+1NxH(m)xH(m−k)ejϕd(m−k)∗=∑m=k+1m≠n;m≠n+kNImxH(m)xH(m−k)ejϕd(m−k)∗+ImxH(n)xH(n−k)ejϕd(n−k)∗+ImxH(n+k)e−jϕd(n)xH∗(n).

It should be pointed out that, as can be seen from Equation (9), the optimization of aperiodic correlation function is considered in this article. Thus, when *k* changes from −P+1 to P−1, $x_{H}\left(n-k\right)$ and $x_{H}\left(n+k\right)$ in Equations (A1) and (A2) may not belong to $x_{H}$. Here, we stipulate that $\left\{x_{p}\left(n\right)\right\}$ are zero when $n\notin \left[1,N\right]$. In addition, for two complex numbers *b* and *d*, the following rules of operations should be kept:

(A3) Re(bd)=Re(b)Re(d)−Im(b)Im(d),

(A4) Re(bd∗)=Re(b)Re(d)+Im(b)Im(d),

(A5) Im(bd)=Im(b)Re(d)+Re(b)Im(d),

(A6) Im(bd∗)=Im(b)Re(d)−Re(b)Im(d).

Hence, Equations (A1) and (A2) can be rewritten as:

(A7) Re(r′HH(k))=∑m=k+1m≠n;m≠n+kNRexH(m)xH(m−k)ejϕd(m−k)∗+RexH(n)RexH(n−k)ejϕd(n−k)+ImxH(n)ImxH(n−k)ejϕd(n−k)+RexH(n+k)e−jϕd(n)RexH(n)+ImxH(n+k)e−jϕd(n)ImxH(n)

and

(A8) Im(r′HH(k))=∑m=k+1m≠n;m≠n+kNImxH(m)xH(m−k)ejϕd(m−k)∗+ImxH(n)RexH(n−k)ejϕd(n−k)−RexH(n)ImxH(n−k)ejϕd(n−k)+ImxH(n+k)e−jϕd(n)RexH(n)−RexH(n+k)e−jϕd(n)ImxH(n).

Based on Equations (A7) and (A8), we can obtain that:

(A9) ∂Re(r′HH(k))∂Re(xH(n))=RexH(n−k)ejϕd(n−k)+RexH(n+k)e−jϕd(n),

(A10) ∂Re(r′HH(k))∂Im(xH(n))=ImxH(n−k)ejϕd(n−k)+ImxH(n+k)e−jϕd(n),

(A11) ∂Im(r′HH(k))∂Re(xH(n))=−ImxH(n−k)ejϕd(n−k)+ImxH(n+k)e−jϕd(n),

(A12) ∂Im(r′HH(k))∂Im(xH(n))=RexH(n−k)ejϕd(n−k)−RexH(n+k)e−jϕd(n).

Similarly, the real and the imaginary parts of ${r^{\prime }}_{\mathrm{HV}}\left(k\right)$ and ${r^{\prime }}_{\mathrm{VH}}\left(k\right)$ can be developed similarly following the Equations (A1)–(A8). Then, the derivatives with respect to the real and the imaginary parts of $x_{H}$ can be obtained as follows:

(A13) ∂Re(r′HV(k))∂Re(xH(n))=RexV(n−k)ejϕd(n−k),

(A14) ∂Re(r′HV(k))∂Im(xH(n))=ImxV(n−k)ejϕd(n−k),

(A15) ∂Im(r′HV(k))∂Re(xH(n))=−ImxV(n−k)ejϕd(n−k),

(A16) ∂Im(r′HV(k))∂Im(xH(n))=RexV(n−k)ejϕd(n−k),

and

(A17) ∂Re(r′VH(k))∂Re(xH(n))=RexV(n+k)e−jϕd(n),

(A18) ∂Re(r′VH(k))∂Im(xH(n))=ImxV(n+k)e−jϕd(n),

(A19) ∂Im(r′VH(k))∂Re(xH(n))=ImxV(n+k)e−jϕd(n),

(A20) ∂Im(r′VH(k))∂Im(xH(n))=−RexV(n+k)e−jϕd(n).

As mentioned before, when k<0, the derivatives of ${r^{\prime }}_{\mathrm{HH}}\left(k\right)$, ${r^{\prime }}_{\mathrm{HV}}\left(k\right)$ and ${r^{\prime }}_{\mathrm{VH}}\left(k\right)$ with respect to the real and the imaginary parts of $x_{H}$ can be obtained by the same procedures from Equations (A1)–(A8). In addition, the results are the same as Equations (A9)–(A20). Then, by combining Equation (21), (A3)–(A6) and (A9)–(A20), the following can be obtained:

(A21) ∂ψ∂Re(xH(n))=2∑k=−P+1P−1Rer′HH(k)xH(n−k)ejϕd(n−k)+Rer′HH(k)xH(n+k)e−jϕd(n)∗+Rer′HV(k)xV(n−k)ejϕd(n−k)+Rer′VH(k)xV(n+k)e−jϕd(n)∗

and

(A22) ∂ψ∂Im(xH(n))=2∑k=−P+1P−1Imr′HH(k)xH(n−k)ejϕd(n−k)−Imr′HH(k)xH(n+k)e−jϕd(n)∗+Imr′HV(k)xV(n−k)ejϕd(n−k)−Imr′VH(k)xV(n+k)e−jϕd(n)∗.
